# Berberine improves insulin resistance in adipocyte models by regulating the methylation of hypoxia-inducible factor-3α

**DOI:** 10.1042/BSR20192059

**Published:** 2019-10-25

**Authors:** Yuanli Wang, Wenwen Gong, Shaofang Lv, Hongmei Qu, Yanling He

**Affiliations:** Department of Obstetrics, Yantai Yuhuangding Hospital, No. 20 Yuhuangding East Road, Zhifu District, Yantai 264000, Shandong Province, China

**Keywords:** Adiponectin, Berberine, Gestational diabetes mellitus, Insulin resistance, Methylation

## Abstract

Methylation of hypoxia-inducible factor-3α (HIF3A) was previously demonstrated to be highly associated with insulin resistance (IR) in patients with gestational diabetes mellitus (GDM). We aimed to study the therapeutic effects of Berberine (BBR) on GDM and the possible mechanisms. The expressions and methylated states of HIF3A in pregnant women with GDM were compared with that in healthy controls. The IR cell models of 3T3-L1 adipocytes was constructed by 1 μmol/l dexamethasone (Dex) and 1 μmol/l insulin (Ins). To evaluate the effects of BBR on IR adipocyte models, cells were subjected to BBR treatment at different concentrations. Transfection of HIF3A siRNA further confirmed the role of HIF3A in the BBR-induced improving effects. Low expression and high methylation of *HIF3A* gene were frequent in the GDM pregnancies. BBR treatment noticeably increased the glucose usage rates, adiponectin secretion and cell differentiation of IR 3T3-L1 adipocytes. Increased HIF3A expression and decreased methylated state of HIF3A were also found in IR adipocytes. Furthermore, HIF3A silencing not only reversed the effects of BBR on improving insulin sensibility, but also partially abolished the expression alterations of insulin-related genes in IR adipocytes induced by BBR treatment. Our results suggest that BBR improves insulin sensibility in IR adipocyte models, and the improving effects of BBR are possibly realized through the inhibition of HIF3A methylation.

## Introduction

As a common medical complication in pregnancy, gestational diabetes mellitus (GDM) is defined as hyperglycemia caused by glucose intolerance and is first detected during pregnancy. Ranging from 2 to 12 %, morbidity of GDM is different in different regions [1,2]. Uncontrolled GDM can lead to multiple adverse complications [3]. For pregnant women with GDM, they were at a high risk of developing eclampsia, hypertension of pregnancy, placental abruption and/or other obstetric complications [4]. In addition, approximately 5% of pregnant women with GDM progressed to type II diabetes mellitus (T2DM) within 26 years after delivery [5,6]. The intrauterine high-glucose environment in GDM pregnancy severely affects the development of the fetus and could cause fetal malformation, macrosomia or other adverse pregnancy outcomes [7]. Moreover, these babies were more likely to develop type II diabetes, childhood obesity or metabolic syndrome [8]. As GDM greatly affects the health of both pregnant women and their fetuses, identifying safe and effective therapeutic methods has become a focus of public health [9].

The pathogenesis of GDM is different from that of type I diabetes, as the former is mainly caused by insulin resistance and adipocyte function impairment [10,11]. DNA methylation can lower the activity of a DNA segment by adding methyl groups to DNA molecule, especially when DNA methylation is located in the gene promoter region such as CpG islands, it functions as a suppressor for gene transcription [2,12,13]. Currently, many studies have shown that DNA methylation was involved in the pathogenesis of GDM [14,15], for instance Ott et al. [16] demonstrated that reducing the expression of insulin receptor by DNA methylation in visceral adipose tissue samples (VAT) contributed greatly to GDM. Furthermore, placenta is known as the critical place for the exchange of oxygen, nutrients, hormones and waste between the circulations of mother and fetus [17], and global placental DNA hypermethylation was also reported to be highly associated with GDM [18].

Hypoxia-inducible factor-3α (HIF3A) belongs to the transcriptional factor family of hypoxia-inducible factors (HIFs) [19]. By regulating different target genes, HIFs family plays an important role in adipose tissue dysfunction, inflammation, even in cancer [20]. HIF3A was demonstrated to be able to promote the metastatic phenotypes in pancreatic cancer [21]. Recently, HIF3A was also reported to be involved in the differentiation of adipose cell [22], and the methylation of HIF3A was highly associated with body mass index (BMI) [23]. Studies demonstrated that the expression and methylation of HIF3A in adipocyte were fat depot-specific and is related to metabolism in adipose tissue and insulin resistance, which are believed as two factors contributing to GDM progression [24,25]. These researches suggest that HIF3A could be used a promising target for the improvement of GDM.

Berberine (BBR) is the isoquinoline alkaloid extracted from *Rhizoma coptidis* [*Coptis chinensis* Franch. (Ranunculaceae)]. BBR possesses a variety of pharmacological effects such as anti-cancer, anti-inflammation, regulating blood glucose and lipid and could treat infectious diarrhea [26–29]. In Chinese medicine, BBR has long been used the treatment of T2DM, high blood glucose and lipid [30]. Currently, studies have increasingly proved that BBR has positive effects on IR, lipid metabolism and diabetes. In 2008, Yi et al. [31] demonstrated that BBR could significantly reverse IR in 3T3-L1 adipocytes induced by free fatty acid by repressing the phosphorylation of IKKβ. Similarly, some recent researches indicated that BBR could significantly promote the active secretion of insulin through stimulating free fatty acid and cascade reaction of insulin-like growth factor-1 (IGF-1) and enhance the sensitivity of liver, muscle and adipose tissues to insulin [32]. Based on these findings, we speculated whether BBR could be able used for the treatment of GDM and aimed to investigate the underlying mechanisms.

## Materials and methods

### Patient data

The present study was reviewed and approved by Yantai Yuhuangding Hospital. A total of 88 pregnant women (45 pregnant women with GDM and 43 healthy controls) treated in our hospital from February 2016 to February 2017 were enrolled. The diagnostic criteria for GDM were as follows: fasting plasma glucose ≥ 100 mg/dl, 1-h oral glucose tolerance test (OGTT) ≥ 180 mg/dl and 2-h OGTT ≥ 155 mg/dl. Subcutaneous adipose tissues (1 cm^3^) were collected from the abdominal region during cesarean delivery and snap-frozen in liquid nitrogen and stored at −80°C for expression analysis. Written informed consent was obtained from each study subject.

### Real-time quantitative PCR

Total RNAs were extracted from the collected subcutaneous adipose tissues and mouse preadipocyte 3T3-L1 cells using TRIzol reagent (Invitrogen, Carlsbad, CA). Reverse transcription of RNA was performed using an iScript cDNA synthesis kit (Bio-Rad Laboratories, Inc, Hercules, CA, U.S.A.). Then, the SYBR Green Master Mix (Applied Biosystems, U.S.A.) was used to determine the relative expression levels of the genes under the reaction conditions as follows: an initial denaturation at 95°C for 30 min, followed by 40 cycles of denaturation at 95°C for 15 s and annealed and extended at 60°C for 30 s. The relative mRNA levels were determined by the 2^−ΔΔ*C*_T_^ methods [33]. β-actin served as a stable internal control. The nucleotide sequences of primers are listed in Table 1.

**Table 1:** The primers for real-time quantitative PCR

Primer name	Species	Sequence (5′–3′)
HIF3A	Human	Forward: 5′-GTCGGAGAGTATCGTCTGTGTC-3′
		Reverse: 5′-TCTGCGAGAGTGTTGCTCCGTT-3′
β-actin	Human	Forward: 5′-GGCTGTATTCCCCTCCATCG-3′
		Reverse: 5′-CCAGTTGGTAACAATGCCATGT-3′
HIF3A	Mouse	Forward: 5′-GCAATGCCTGGTGCTTATCT-3′
		Reverse: 5′-TCCTCTCGTCGCAGTATGTG-3
HIF1A	Mouse	Forward: 5′-TTCCAGTTACGTTCCTTCGATCA-3′
		Reverse: 5′-TTTGAGGACTTGCGCTTTCA-3
HIF2A	Mouse	Forward: 5′-GTGCTCCCACGGCCTGTA-3′
		Reverse: 5′-TTGTCACACCTATGGCATATCACA-3
IRS-1	Mouse	Forward: 5′-AAGGAGTCGGCTCCAGTGT-3′
		Reverse: 5′-AGAGGGGCAGTCCTGAGAGT-3
GLUT4	Mouse	Forward: 5′-GACGGACACTCCATCTGTTG-3′
		Reverse: 5′-GCCACGATGGAGACATAGC-3′
β-actin	Mouse	Forward: 5′-CCTGTACGCCAACACAGTGC-3′
		Reverse: 5′-ATACTCCTGCTTGCTGATCC-3′

Abbreviations: GLUT4, glucose transporter 4; IRS-1, insulin receptor substrate 1.

### Methylation-specific PCR

The subcutaneous adipose tissues collected from three GDM women and three normal pregnant women were selected for determining the methylation state of HIF3A. Total DNA from subcutaneous adipose tissues was extracted by Maxwell 16 Tissue DNA Purification Kit (Promega, Madison, WI, U.S.A.) and the quantity of isolated DNA samples was measured as previously described [34]. The isolated DNA samples (500 ng) were subjected to bisulfite treatment by fast bisulfite kit (Qiagen, Frankfurt, Germany) following the manufacturer’s instructions. The bisulfite-induced unmethylated cytosine residues convert into uracil without affecting the 5-methylcytosine residue, which helps observe the methylation status of the target DNA segment.

The methylation states in the subcutaneous adipose tissues of GDM and normal groups were determined by methylation-specific PCR (MSP). The MethPrimer design tool (http://www.urogene.org/Methprimer/index1.html) was used to design the specific primers for the determining the methylated and unmethylated status of the promoter segment. The primer sequences were Left primer: 5′-TAAGGGTTTTAAATTTGGAGTTAAT-3′, Right primer: 5′-AAAATAAAAAAACAATATTCCTTCC-3′. Fifty microliters of MSP mixture contained 1× polymerase chain reaction buffer (15 mmol/l MgCl_2_), 2.5 mmol/l mixture of dNTPs, 10 pM of each primer, 4 U HotStart Taq DNA polymerase (Qiagen, Frankfurt, Germany) and 25–50 ng of bisulfite-modified DNA, and the reaction parameters were as follows: an initial denaturation at 95°C for 15 min, followed by 40 cycles at 94°C for 30 s, 60°C for 1 min and 72°C for 30 s, finally extended at 75°C for 3 min. MSP products were visualized by 2% agarose gel electrophoresis and Ethidium Bromide staining.

### Cell culture and differentiation

Mouse preadipocyte 3T3-L1 cell line was purchased from American Type Culture Collection (ATCC, Manassas, VA, U.S.A.). Cells were cultured into 48-well plates (5 × 10^5^ cells/well) in Dulbecco’s modified Eagle’s medium (DMEM, ATCC) containing 10% fetal bovine serum (FBS, ATCC), 2 mmol/l glutamine and 20 mmol/l HEPES (pH 7.4) in 5% CO_2_ at 37°C for 2 days until 70% confluence. For cell differentiation, 3T3-L1 cells were incubated in high-glucose DMEM containing 4.5 g/l glucose, 0.5 mmol/l 3-isobutyl-1-methylxanthine (IBMX), 1 μmol/l dexamethasone (Dex, Solarbio, Beijing, China) and 1.67 μmol/l insulin (Ins, Solarbio) and 10% FBS for 3 days. Then, the cells were transferred to differentiation maintenance medium supplemented with 10% FBS and 0.41 μmol/l Ins for 3 days. After 4-day incubation in the growing medium (DMEM and 10% FBS), mature 3T3-L1 adipocytes were achieved.

### Oil Red O staining

Oil Red O staining was used to confirm the mature 3T3-L1 adipocytes. In brief, the mature 3T3-L1 cells were fixed with ice-cold acetone for 30 min and then stained with 0.3% Oil Red O (Sigma–Aldrich, Darmstadt, Germany)/60% isopropanol solution (Invitrogen) for 2 h. The stained lipid droplets were observed under phase contrast microscopy (Nikon Corporation, Tokyo, Japan).

### Establishing IR adipocyte models

In order to establish IR adipocyte models, the mature 3T3-L1 adipocytes were divided into four groups, which were Control, Dex, Dex+Ins1 and Dex+Ins10 groups. In Control, cells were cultured with Phenol Red-free DMEM (BioWiseTech, Co., Ltd., Suzhou, China). Cells in the Dex group were cultured in Phenol Red-free DMEM containing 1 μmol/l Dex. Mediums in Dex+Ins1 and Dex+Ins10 groups were supplemented with 1 and 10 μg/ml Ins. All group cells were incubated in 5% CO_2_ at 37°C for 3 days until the IR adipocyte model was successfully established. In IR adipocyte models, to evaluate the effects of different conditional medium induced by IR on the frequency of using glucose, we measured the glucose contents in the culture supernatant of each group by glucose oxidase and peroxidase (GOD-POD) method (Agape Diagnostic Kits, Ernakulam, Kerala). GOD-POD assay was performed by Roche Hitachi P800 auto-analyser (Roche Diagnostics GmbH, Mannheim).

### Cell treatment

Based on the effects of BBR on enhancing insulin sensibility of adipose tissues, we used BBR to treat IR adipocyte models. The cells were incubated in 48-well plates (5 × 10^5^ cells/well) containing Phenol Red-free DMEM with 4.5 g/l glucose. Here, the cells were divided into six groups: Control, Model, rosiglitazone (ROZ), BBR-L, MMR-M and BBR-H groups. Except for the Control group, the mediums of all groups were supplemented with 1 μg/ml Ins and 1 μmol/l Dex for 3-day induction of IR adipocyte model. Next, 20 μmol/l ROZ was added into the culture medium of rosiglitazone group (ROZ), while the culture mediums in BBR-L, BBR-M and BBR-H groups were respectively supplemented with low, middle and high concentrations (1, 10 and 100 μmol/l) of BBR [35]. The ROZ group served as the positive control for the evaluation of the effects of BBR on the IR. After incubating the cells in 5% CO_2_ at 37°C for 3 days, the cells from all groups were harvested for subsequent experiments.

### Cell transfection

To assess the role of HIF3A in the decrease in IR by BBR in IR adipocyte models, HIF3A siRNA (siHIF3A: Sense: 5′-GGAGACAGAUCUAGAUAUA-3′, Antisense: 5′-CCUCUGUCUAGAUCUAUAU-3′) synthesized by GenePharma (Shanghai GenePharma Co., Ltd., Shanghai, China) was used for cell transfection. IR differentiated 3T3-L1 adipocytes were seeded into 24-well plates (1 × 10^5^ cells/well) at 37°C. siHIF3A or siNC (50 nM) was transfected into the adipocytes using Lipofectamine™ RNAiMAX Transfection Reagent (Invitrogen). Forty-eight hours after the transfection, the cells were harvested for subsequent experiments. The transfection efficiency was determined by performing real-time quantitative PCR (RT-qPCR) and Western blot.

### Glucose consumption assay

To determine the glucose usage rates of IR 3T3-L1 adipocytes, we detected the glucose concentration in the culture medium by GOD-POD method. After 3-day incubation, culture supernatant was separated by centrifuging at 4000×***g*** at 4°C for 15 min. Glucose levels were determined by GOD-POD assay (Agape Diagnostic Kits, Ernakulam) using Roche Hitachi P800 auto-analyser (Roche Diagnostics GmbH). The glucose usage rates (%) = (1 – Glucose content_(experimental groups)_/Glucose content _(IR model groups)_) × 100%.

### Adiponectin secretion

After 3-day incubation, cell-free supernatants were obtained by centrifuging at 5000×***g*** at 4°C for 15 min. The concentrations of adiponect in each medium sample were measured using a mouse adiponectin Enzyme-linked immunosorbent assay (ELISA, Otsuka Pharmaceuticals, Tokyo, Japan). Briefly, the supernatants were transferred to microwell plates coated with antibodies against adiponectin and incubated with the biotin-labeled secondary antibody. A streptavidin horseradish–peroxidase conjugate was then added into the plates. Tetramethylbenzidine/peroxide served as the substrate. The adiponectin products were analyzed using a scanning multiwell spectrophotometer (ELISA reader MR 5000, Dynatech, Guernsey, U.K.) at 450 nm.

### Western blot

Total protein isolated from 3T3-L1 cells from each experimental group were performed using RIPA buffer (Beyotime Biotechnology, Shanghai, China). Then, 30 μg proteins from each group were subjected to 10% SDS/polyacrylamide gel and then transferred to polyvinylidene fluoride membranes (Bio-Rad Laboratories, Inc, CA, U.S.A.). The membranes were blocked for 1 h in 5% fat-free milk at 37°C and incubated with primary antibody against HIF1A (#ab1, 1:1000, 120 kDa), HIF2A (#ab199, 1:1000, 118 kDa), HIF3A (#ab2165, 1:2000, 42 kDa), Insulin Receptor Substrate 1 (IRS-1, #ab52167, 1:1000, 132 kDa) and glucose transporter 4 (GLUT4, #ab654, 1:2000, 458 kDa) overnight at 4°C with gentle agitation. Next, the membranes were incubated with horseradish peroxidase–conjugated secondary antibody (1:20000, #ab205718 and #ab205719, 42 and 52 kDa, Abcam) for 1 h at room temperature and developed using an enhanced chemiluminescence kit (Amersham Life Sciences, U.K.). β-actin (#ab8226, 1:5000, 42 kDa) served as an internal control. All primary antibodies were purchased from Abcam (Cambridge, MA, U.S.A.).

### Statistical analysis

The results were shown as means ± SD. The analysis was conducted using GraphPad Prism 6. One-way ANOVA followed by a post-hoc Tukey’s test was carried out for comparisons among three or more groups. The main factors such as dosage used and duration of time were significant different among the groups. *P*<0.05 was considered as statistically significant.

## Results

### Low expression and high methylation of *HIF3A* gene were common in pregnant women with GDM

**Figure 1:**
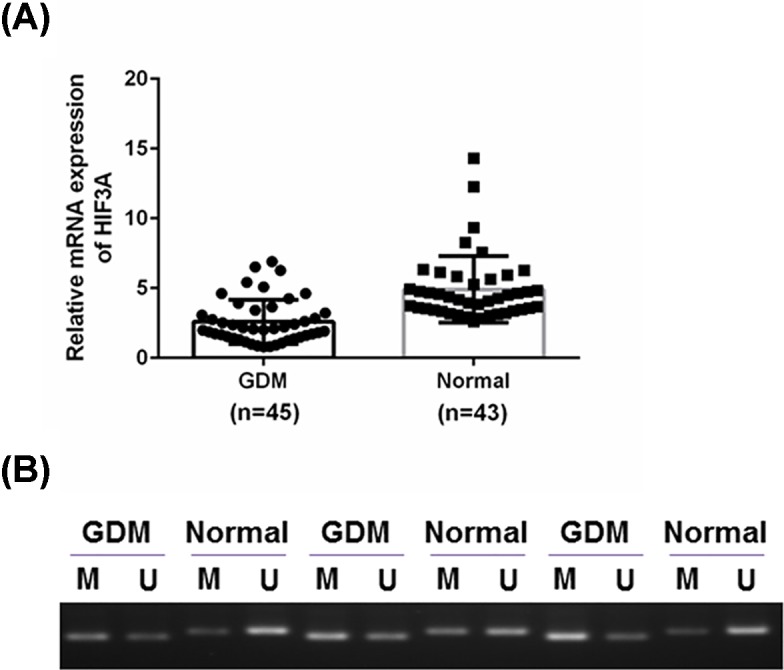
Low expression and high methylation of HIF3A were frequent in the GDM pregnant women We compared the expression and methylation of HIF3A in the subcutaneous adipose tissues from GDM patients with that from healthy pregnant women. (**A**) A total of 88 subcutaneous adipose tissues were obtained from 45 GDM and 43 healthy pregnant women. We compared the HIF3A mRNA levels of the GDM group with that of the normal group. (**B**) Three random samples were chosen from each group, we then measured the methylated and unmethylated states of *HIF3A* gene in this chosen sample. Results were presented as means ± SD. β-actin served as the loading control.

The anthropometric features of the women involved were demonstrated in Table 2. Using the Mann–Whitney U test, no statistical differences in these variables, including age, BMI (kg/m^2^) and body fat (%) were observed between the two groups. First, we compared the mRNA levels of HIF3A in the subcutaneous adipose tissues between the GDM and healthy pregnant women, and found that low-expressed HIF3A was common in the GDM group, compared with that in the normal group (Figure 1A). Then, three GDM and three normal samples were randomly chosen for determining the methylation state by MSP. As shown in Figure 1B, the level of methylated *HIF3A* gene were higher than the level of methylated HIF3A in GDM group, while subcutaneous adipose tissues of the normal group had a higher level of unmethylated HIF3A in comparison with that of methylated HIF3A. However, it would be helpful for using quantification chart of methylation, which would be considered in future study.

**Table 2:** Patient characteristics

Samples	*n*	Age (Mean ± SD)	BMI (kg/m^2^) (Mean ± SD)	Body fat (%)
Pregnant women with GDM	45	39 ± 3.98	31.29 ± 2.56	40.2 ±3.32
Pregnant women without GDM	43	37 ± 3.15	32.56 ± 1.67	39.3 ± 2.15

### Establishment of IR 3T3-L1 adipocyte model

**Figure 2:**
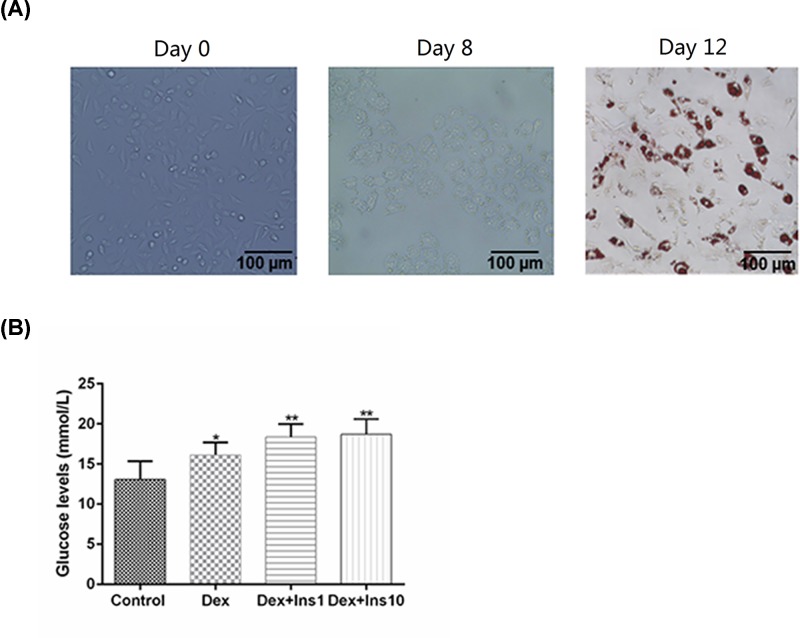
Establishment of IR 3T3-L1 adipocyte model 3T3-L1 preadipocytes were chosen as the experimental subject for the establishment of the IR model. (**A**) A light microscope and Oil Red O staining were used for the observation of adipocyte differentiation. (**B**) The glucose levels in the culture supernatants were determined by GOD-POD method to reflect the ability of adipocytes resistance to insulin under different IR inducing induction programs. Results were presented as means ± SD. *P<0.05, **P<0.01 vs. Control.

As listed in Figure 2A, the cellular morphology of 3T3-L1 adipocytes was observed using a light microscope. The undifferentiated adipocytes showed a fusiform shape (day 0). After the induction of differentiation, lipid droplets accumulated gradually and increased as cell differentiation (day 8) progressed. At day 12, the cells were subjected to Oil Red O staining. The number of lipid droplets can reflect the differentiation of adipocytes. The IR adipocyte model was induced by three types of IR-induced conditional medium. As listed in Figure 2B, the IR 3T3-L1 adipocyte model induced by the combination of Dex and Ins had a lower glucose usage rate, compared with that in Dex group. No observable differences in the glucose levels between the Dex+Ins1 and Dex+Ins10 groups were observed, therefore, Dex 1 μmol/l and Ins 1 μg/ml were used to establish IR adipocyte model in the subsequent experiments.

### BBR had the ability to improve the IR in the IR adipocyte model

**Figure 3:**
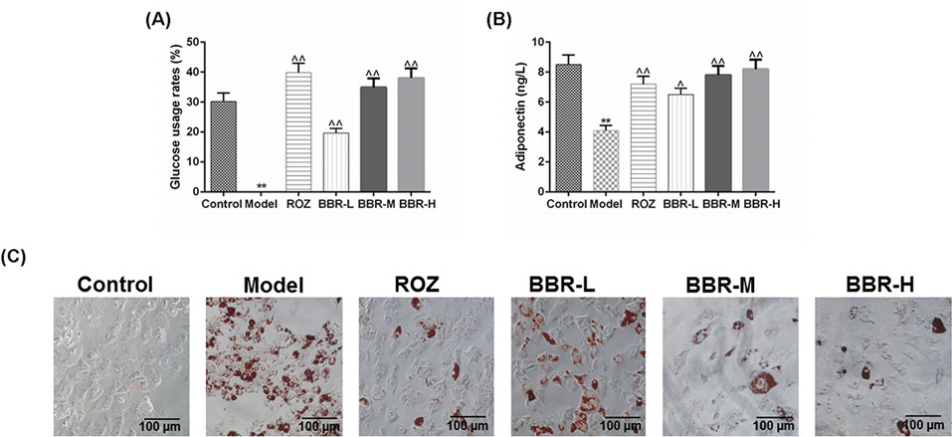
BBR had the ability to improve the IR in the IR adipocyte model To evaluate the effects of BBR on the insulin sensibility of IR adipocyte models, three different concentrations of BBR were introduced to treat IR adipocytes. The treatment of ROZ served as a positive control for the evaluation of the effects of BBR on insulin sensibility of IR 3T3-L1 adipocytes. (**A**) The glucose usage rates were determined using GOD-POD method. (**B**) The changes in adiponectin secretion of each group were measured by a mouse adiponectin ELISA kit. (**C**) The changes in lipid droplet number determined by Oil Red O staining reflected the effects of BBR on fatty deposits in 3T3-L1 adipocytes. Results were presented as means ± SD. ^∧^*P*<0.05, ^∧∧^*P*<0.01 vs. Model, **P<0.01 vs. Control.

In order to evaluate the effects of BBR on the insulin sensibility of IR adipocyte models, three different concentrations of BBR were introduced to treat IR adipocytes. The treatment of ROZ was considered as a positive control. We first measured the changes in the glucose usage rates under the effects of BBR or ROZ treatment (Figure 3A), and observed that in comparison with the Model group, the treatment of ROZ and BBR could notably increase the glucose usage rates in IR adipocytes (*P*<0.01). BBR with the medium concentration greatly increased the glucose usage rates, compared with that in BBR-L group. The adiponectin secretion of IR adipocytes was notably decreased, compared with the control (*P*<0.01, Figure 3B). Both ROZ and BBR could significantly increase the secretion of adiponectin in IR adipocyte models, which may contribute to the improvement of IR. BBR at a medium concentration showed a more significant effect on increasing the adiponectin secretion of IR 3T3-L1 adipocytes, compared with that by low concentration of BBR. We also observed the changes in lipid droplet number by Oil Red O staining, as demonstrated in Figure 3C, ROZ and BBR could remarkably inhibit the fatty deposits of 3T3-L1, and the inhibitory effects of medium and high concentrations of BBR on fatty deposits were more significant than that by low concentrations.

### BBR enhanced the expression of HIF3A and down-regulated the level of methylated HIF3A in IR 3T3-L1 adipocytes

**Figure 4:**
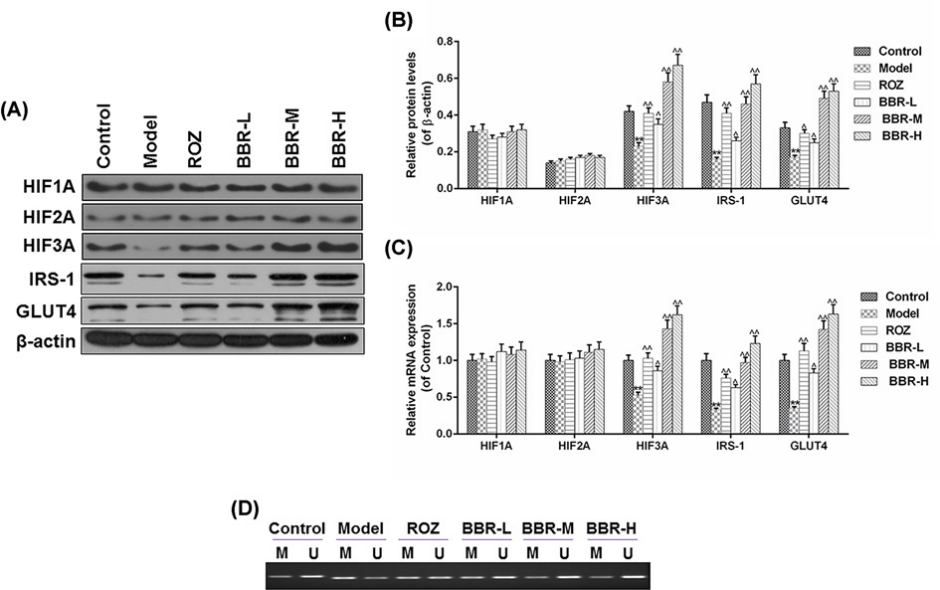
BBR enhanced the expression of HIF3A and decreased the level of methylated HIF3A in IR 3T3-L1 adipocytes (**A**–**C**) We further measured the changes in mRNA and protein levels of HIFs family, IRS-1, and GLUT4 in the IR adipocytes under the effects of ROZ and BBR treatment. (**D**) The effects of ROZ and BBR on the methylated and unmethylated states of HIF3A in IR adipocytes were analyzed using MSP. Results were presented as means ± SD. β-actin served as the loading control. ^∧^*P*<0.05, ^∧∧^*P*<0.01 vs. Model, **P<0.01 vs. Control.

We further measured the expressions of HIFs family, IRS-1 and GLUT4 in the IR adipocytes under the effects of ROZ and BBR treatment. As shown in Figure 4A–C, there were no notable differences in the mRNA and protein levels of HIF1A and HIF2A among different groups. In comparison with the Control group, the expressions of HIF3A, IRS-1 and GLUT4 decreased noticeably in Model group (*P*<0.01). The treatment of BBR at a low concentration could remarkably up-regulate the expression levels of these genes in IR adipocyte models (*P*<0.01). Compared with that in BBR-L group, the medium concentration of BBR showed a more significant effect on the mRNA and protein levels of HIF3A, IRS-1 and GLUT4. Furthermore, we also detected the changes in the methylated and unmethylated states of HIF3A in IR adipocytes under the effects of ROZ and BBR. As shown in Figure 4D, compared with the Control group, the methylated level of HIF3A was higher than the unmethylated HIF3A level in Model group, and ROZ and BBR could markedly reduce the methylated state of HIF3A and up-regulated the level of unmethylated HIF3A in IR adipocytes. Moreover, the effects on the methylated state of HIF3A in BBR-M were more significant than that in BBR-L group, however, no observable differences were found in BBR-M and BBR-H groups. Taken together, the medium concentration of BBR was sufficient to reverse the methylated state of HIF3A as well as the expressions of IRS-1 and GLUT4 in IR adipocytes.

### siHIF3A reversed the improving effects of BBR on insulin sensibility of IR 3T3-L1 adipocyte models

**Figure 5:**
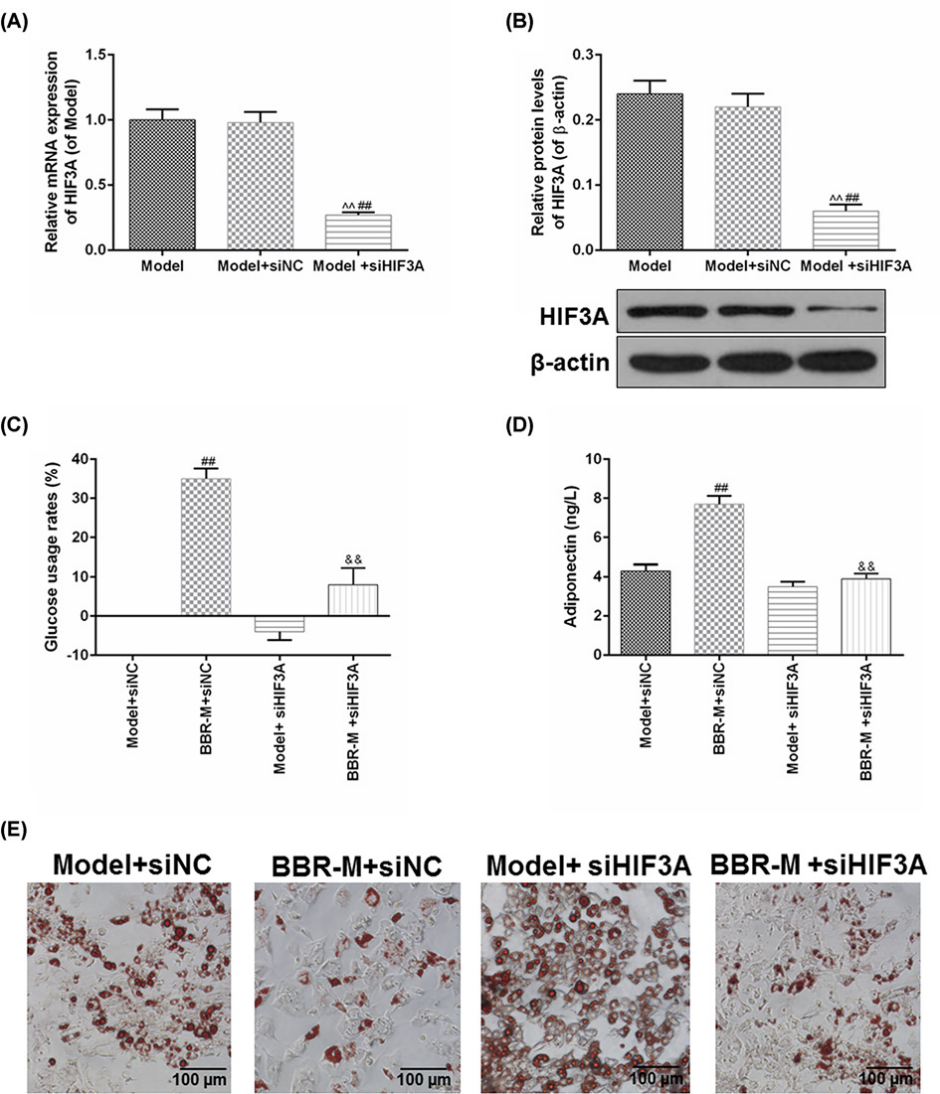
siHIF3A reversed the improving effects of BBR on insulin sensibility of IR 3T3-L1 adipocyte models To further confirm the role of HIF3A in the BBR-induced the IR improvement, siHIF3A was used to regulate the expression of HIF3A in the IR adipocyte models. (**A**,**B**) The transfection efficiency of siHIF3A was measured by RT-qPCR and Western blot. (**C**) The rates of glucose usage were determined by GOD-POD method. (**D**) The effects of siHIF3A silencing on the adiponectin secretion were analyzed by a mouse adiponectin ELISA kit. (**E**) Oil Red O staining was applied to measure the changes in the fatty deposits of IR adipocytes with the effects of HIF3A silencing and BBR. Results were presented as means ± SD. β-actin served as the loading control. ^∧∧^*P*<0.01 vs. Model; ^##^*P*<0.01 vs. Model+siNC; ^&&^*P*<0.01 vs. BBR+siNC.

To further confirm the role of HIF3A in BBR-induced the IR improvement, siHIF3A was used to regulate the expression of HIF3A in the IR adipocyte models. In Figure 5A,B, the transfection of siHIF3A could effectively down-regulate the mRNA and protein levels of HIF3A in the IR adipocyte models, compared with the Model and Model+siNC groups (*P*<0.01). The introduction of siHIF3A could further enhance IR adipocyte models, however, HIF3A silencing not only decreased the glucose usage rate in IR adipocytes, but also significantly inhibited the improving effects of BBR on the glucose usage rate in IR adipocytes (*P*<0.01, Figure 5C). Meanwhile, increased adiponectin induced by BBR treatment also decreased notably under the effects of HIF3A silent (*P*<0.01, Figure 5D). Moreover, we also observed the changes in the number of lipid droplets in the IR adipocyte models (Figure 5E), and found that the HIF3A silent could not only promote the fatty deposits, but also observably reverse the inhibitory effects of BBR on fatty deposits in IR adipocyte models. Collectively, these results suggested that the improving effects of BBR on the insulin sensibility of IR adipocyte models may be realized by the regulation of HIF3A.

### HIF3A silencing reversed the regulatory effects of BBR on the insulin-related gene in IR adipocytes

**Figure 6:**
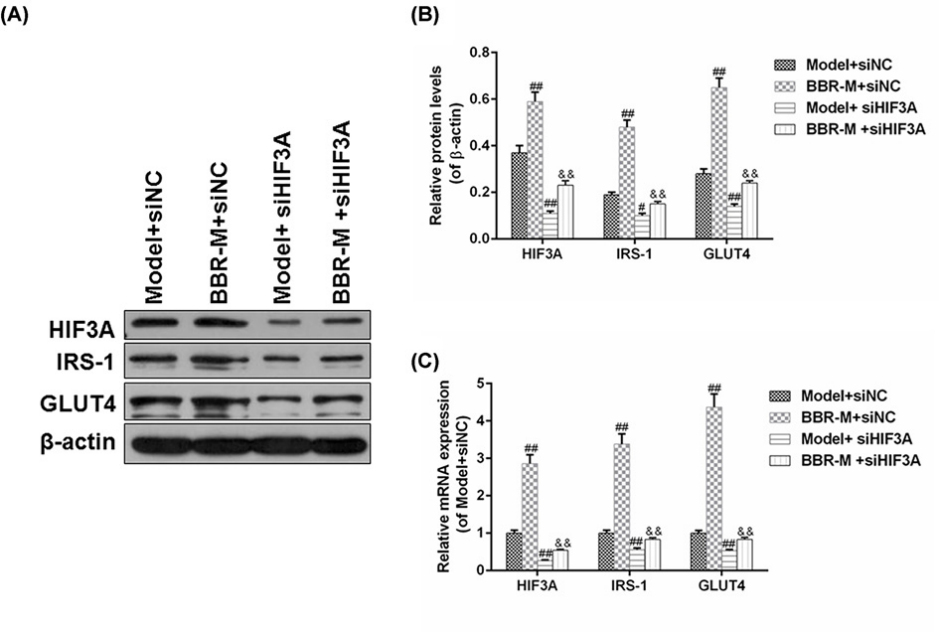
HIF3A silencing reversed the regulatory effects of BBR on the insulin-related gene in IR adipocytes We also detected the changes in the mRNA and protein levels of HIF3A, IRS-1, and GLUT4 in IR adipocyte models under the effects of BBR and HIF3A silencing. (**A**,**B**) The effects of HIF3A silencing and BBR on the protein level of HIF3A, IRS-1 and GLUT4 in IR adipocyte models were analyzed by Western blot. (**C**) The changes in mRNA levels were determined by RT-qPCR. Results were presented as means ± SD. β-actin served as the loading control. ^#^*P*<0.05 and ^##^*P*<0.01 vs. Model+siNC; ^&&^*P*<0.01 vs. BBR+siNC.

The changes in the mRNA and protein levels of HIF3A, IRS-1 and GLUT4 in IR adipocyte models were detected under the effects of BBR and HIF3A silencing. As shown in Figure 6A,B, HIF3A silencing inhibited the protein levels of HIF3A, IRS-1 and GLUT4 in IR adipocyte models and also effectively reversed the up-regulated protein levels of HIF3A, IRS-1 and GLUT4 protein levels (*P*<0.01) induced by BBR. The tendencies of mRNA changes in these genes were basically consistent with those at protein levels (Figure 6C).

## Discussion

In this current study, our findings demonstrated that BBR showed the ability to improve insulin sensibility in IR adipocyte models. Based on the expression analysis of the clinical subcutaneous adipose tissues of GDM patients, we found that low expression of HIF3A was frequent in pregnant women with GDM. Meanwhile, MSP showed that the methylated state of *HIF3A* gene in GDM patients was higher than that in healthy pregnant women, which could explain the low expression of HIF3A observed in GDM patients. A previous study has proved a high correlation of the methylation of *HIF3A* gene with the IR in GDM [36]. Therefore, HIF3A might be a promising target for improving IR in GDM. The IR cell model was constructed based on 3T3-L1 adipocytes, which were widely used for investigating IR [37,38]. After the treatment of BBR, the frequency of using glucose and adiponectin secretion in IR adipocyte models were both notably increased, while the fatty deposits were much reduced. The subsequent expression analysis showed that BBR could observably up-regulate the expression of HIF3A in IR models by reducing the methylated state of HIF3A, and that IRS-1 and GLUT4 expressions were positively correlated with the concentrations of BBR. SiHIF3A was used to negatively regulate the expression of HIF3A in IR adipocytes in order to further confirm whether BBR-induced IR improvement was mediated through up-regulating the expression of HIF3A in IR adipocyte models, and we observed that HIF3A silencing could effectively reverse BBR-induced improving effects on insulin sensibility. Taken together, our results indicated that BBR showed a potently improving ability in the insulin sensibility of IR adipocyte models, and such effects of BBR on IR model may rely on the reduction in HIF3A methylation and the increased expression of HIF3A.

Adipose tissue primarily consists of adipocytes and it functions as a fat reservoir. In addition, as the largest endocrinal organ, cytokines produced by adipocytes (collectively called adipocytokines) in adipose tissue could participate in various metabolisms including glucose and lipid metabolism [39,40]. The previous study showed that the abnormal secretion of adipocytokines play an important role in the progression of GDM [41]. Adiponectin, an adipocyte-specific adipocytokine, is known for its ability to improve the glucose profile through enhancing insulin sensibility and secretion or modulating liver and muscle-mediated glucose metabolism [42,43]. In 2001, Yamauchi et al. [44] demonstrated that the down-regulation of adiponectin was involved in IR development, and that enhancing adiponectin could effectively improve IR and T2DM. Adiponectin-knockout pregnant mice almost inevitably develop glucose intolerance and IR in the late stages of pregnancy, however, the administration of adiponectin had a notably improving effect on glucose intolerance and IR [45]. These researches indicated that the reduction in adiponectin is accompanied by IR, and these findings were consistent with our data that the level of adiponectin was much reduced in the IR 3T3-L1 adipocyte models. After the treatment of BBR, the noticeable up-regulation of adiponectin, in turn reduced the glucose utilization of IR adipocytes. Furthermore, IRS-1 has been demonstrated to play a critical role in the insulin-induced metabolism, and the knockdown of IRS-1 can lead to IR in the insulin-sensitive skeletal muscle, adipose tissue and liver [46,47]. The down-regulation of GLUT4 and IRS-1 in adipose tissues was also reported to be associated with the development of GDM [48]. In 2017, Luna-Vital et al. [49] demonstrated that anthocyanins isolated from colored corn could evidently improve insulin sensibility via promoting the phosphorylation of IRS-1 induced by insulin, which, subsequently, enhanced GLUT4 translocation and glucose uptake in 3T3-L1 adipocytes. In our study, the expressions of IRS-1 and GLUT4 remarkably decreased in IR adipocyte models. The positive effects of BBR on the expression of IRS-1 and GLUT4 also suggested the potential ability of BBR in improving insulin sensibility of IR adipocyte models. Taken together, our results suggested that through improving insulin sensibility by the promotion of adiponectin secretion and the activation of insulin signaling, BBR could be used as a promising agent for the treatment of GDM.

Furthermore, our study suggested that the improving effects of BBR on the insulin sensibility of IR adipocyte models might be dependent on the regulation of DNA methylation of HIF3A. Studies have proved the potential role of DNA methylation in the development of GDM [14,50], for example, Ott et al. [16] demonstrated that the methylation of insulin receptor promoter in adipose tissues contributes critically to the GDM pathophysiology, especially in VATs. HIF3A is usually highly expressed in the adipocytes, and the up-regulation of HIF3A is a typical response to hypoglycemia and glucoprivation *in vivo* [51]. Meanwhile, the aberrant expression of HIF3A could noticeably induce the expressions of multiple adipocytokines and accelerate adipose differentiation [22]. These researches indicated that HIF3A also played a potential role in the regulation of glucose metabolism in adipocytes. Several recent studies showed that the expression and methylation of HIF3A in adipose tissues were important in insulin sensibility and functions of adipose tissues [25,52]. Moreover, Zhang et al. [36] proved a high correlation of methylation of HIF3 to the IR development in GDM. All these researches suggested the potential value of HIF3A methylation in the improvement of GDM. In this current study, we found that the improving effects of BBR on the IR adipocyte models were accompanied by the up-regulated expression level and the reduced methylated state of HIF3A, however, the transfection of siHIF3A could noticeably reverse the positive effects of BBR. Taken together, our data suggested that the improving effects of BBR on the IR adipocyte models were mainly dependent on the regulation of HIF3A expression and methylation.

## Conclusion

To conclude, the high methylation and low expression of HIF3A were frequent in pregnant women with GDM. Our results showed that the treatment of BBR could notably increase the glucose utilization, adiponectin secretion and cell differentiation of IR adipocytes, which were accompanied by the up-regulation of HIF3A and the reduction in methylated state of HIF3A. However, HIF3A silencing could significantly reverse the BBR-induced positive effects on IR adipocyte models. Collectively, our study indicated that BBR is a promising therapeutic drug for improving the IR in GDM through regulating HIF3A methylation and expression.

## Abbreviations

ATCC: American Type Culture Collection
BBR: berberine
BMI: body mass index
CpG: Cytosine-phosphate-Guanine
Dex: dexamethasone
DMEM: Dulbecco’s modified Eagle’s medium
ELISA: enzyme-linked immunosorbent assay
FBS: fetal bovine serum
GDM: gestational diabetes mellitus
GLUT4: glucose transporter 4
GOD-POD: glucose oxidase and peroxidase
HIF: hypoxia-inducible factor
HIF3A: hypoxia-inducible factor-3α
IKK: inhibitor of nuclear factor kappa kinase
Ins: insulin
IRS-1: insulin receptor substrate 1
MSP: methylation-specific PCR
OGTT: oral glucose tolerance test
RIPA: Radio-Immunoprecipitation Assay
ROZ: rosiglitazone
T2DM: type II diabetes mellitus
VAT: visceral adipose tissue
